# Differences in Predictions for Survival and Expectations for Goals of Care between Physicians and Family Surrogate Decision Makers of Chronically Critically Ill Adults

**Published:** 2017-11-24

**Authors:** Sara L Douglas, Barbara J Daly, Amy R Lipson

**Affiliations:** School of Nursing, Case Western Reserve University, USA

**Keywords:** Goals of care, Critical care, Mortality prediction

## Abstract

The purpose of this study was to determine the accuracy and concordance between physicians (MDs) and family surrogate decision makers (FSDMs) in predicting 3 month post-hospital patient mortality and concordance in identifying patient goals of care. A prospective cohort study was conducted in 3 intensive care units (ICUs). Two-hundred and sixty-four FSDMs and 54 attending MDs of patients who had resided in the ICU for >3 days were enrolled in the study. Expectation for mortality was measured dichotomously and goals of care were measured using a continuous visual analog scale. A value of 50 represented equal weight placed on goals of survival and QOL. Both MDs and FSDMs had mortality predictions that were lower than actual mortality. For MDs and FSDMs, their mortality predictions were most accurate at study enrollment. Discordance between MD and FSDM goals of care ranged from 36.4% at enrollment to 55.4% 15 days later (p=0.003). Our findings of optimistic prognosis for survival are consistent with the work of others. Our high rate of discordance regarding goals of care provided support for the need to establish standard processes to assure that values of patients and families are solicited and incorporated into treatment discussions for long-stay ICU patients.

## INTRODUCTION

Among the many challenges in providing excellent end of life (EOL) care, the process of transitioning from acute cure or survival-oriented plans of care to comfort-oriented plans is among the most complex. There is strong national consensus about the great need to discover approaches to improve the delivery of end of life (EOL) care in the ICU^[1–3]^. A particularly relevant subgroup of ICU patients is those who experience an acute crisis and progress to chronic critical illness (CCI). CCI patients are characterized by lengthy ICU stays and prolonged mechanical ventilation with poor outcomes. In-hospital mortality is ~30% with additional 4 month post-hospital mortality being 25%; those who survive are at great risk for significant decrease in quality of life (QOL)^[4–6]^.

Due to the severity of their illness, CCI patients usually require family surrogates (FSDM) to make their medical decisions thus presenting unique and complex challenges in provider-family communication as well as challenges in evaluating treatment effectiveness and transitioning to a palliative or EOL focus. By 2020, it is estimated that there will be >600,000 CCI patients^[7,8]^. While their care is costly in financial terms, the human toll on both patients and their family members is equally significant^[9,10]^.

Research has established that effective provider-family communication influences treatment decisions, yet interventions focused upon communication have had little effect^[11,12]^. Despite decades of study and tests of interventions targeting specific elements, there continue to be concerns about prolonged use of ineffective therapies, resulting in suffering of both patients and families, as well as moral distress of clinicians. The situation of CCI, with the complex interface of acute-on-chronic conditions and uncertain prognoses, presents enormous challenges to clinicians and family surrogates in attempting to assess treatment effectiveness and identify the appropriate criteria and time for transitioning to a focus on palliation and EOL measures.

In particular, consideration and evaluation of changing goals of care require that the primary treatment team recognize the probability of poor outcomes and need for explicit discussion with families of the expected benefits and burdens of alternative treatment plans that do not focus solely upon survival at all cost. Prognostication is thus an essential component of supporting the transition process and is particularly challenging for those caring for CCI patients. The CCI, by definition, are those patients who have not followed the usual pattern of short stays and rapid improvement following a crisis. The co-morbidity burden is high, making predictions based solely on primary diagnosis invalid.

Interviews with family surrogates have confirmed that families want and rely on prognostic estimates from physicians, even while recognizing inevitable uncertainty^[13–17]^. Physician prediction of ICU mortality has been studied extensively and evidence suggests that MDs are relatively accurate in predicting ICU survival for “traditional” ICU patients but little is known about prognostication of MDs or family surrogate decision makers for CCI patients. This is important to ascertain as the presence of unrealistic long-term outcome expectations by either healthcare personnel or family can be a major impediment to treatment decision making and transitioning to an EOL care plan. In addition, discordance in goals and expectations between MD and family surrogate decision makers can exacerbate this impediment^[18,19]^.

The focus of this article is upon the examination of factors that can be a major influence on discussions of treatment transitions: (a) MD and family surrogate prognostication regarding likelihood of patient death over time, and (b) concordance between MD and family surrogate perceptions of goals of care over time in the ICU. Based upon prior research, concordance between physician and family surrogate expectations for long-term outcomes (survival, quality of life etc.) are key aspects of perceptions of goals of care and in ensuring meaningful discussions regarding treatment decisions^[20–22]^. Agreement not only about the goals of care but expectations for key outcomes is essential for ensuring treatment decisions that are founded on shared perceptions and understandings of prognosis^[21,23]^.

## METHODS

### Design

This was a descriptive longitudinal correlational study conducted from February, 2012 to June, 2014. Physician (MD) critical care intensivists and family surrogates (FSDM) of eligible patients were surveyed regarding values, expectations, and evaluation of treatment effectiveness on the 3rd–5th day of the ICU patient’s stay, and every 5 days until ICU discharge or death. At each time point the focus of care was documented from the medical record. The study was approved by the Institutional Review Board of University Hospitals Cleveland Medical Center. Details about the parent study have been reported previously^[24]^.

### Participants

This study took place in three adult intensive care units (medical, surgical and neuroscience) in University Hospitals Cleveland Medical Center, a 950 bed tertiary academic institution. All ICUs were staffed by intensivists, fellows, residents, and nurse practitioners. Surgeons retained primary responsibility for their patients, but relied on the intensivists for managing day to day care. The intensivists with primary responsibilities for day-to-day care of the patients were interviewed for the study.

A convenience sample was employed. Family surrogates were eligible for enrollment if they were the identified decision maker for a patient who lacked capacity for decision making (Glasgow eye score<3 or Glasgow motor score<6) and who was not expected to be discharged from the ICU within the next 48 h. In addition, the surrogate had to be over 18 years of age, English-speaking, and available for interviews. Although we were primarily interested in decisions for patients who progressed to states of chronic critical illness, we sought to identify patients who were not following the more typical trajectory of two-three day stays early in their ICU course in order to examine changes over time from the start of their stay.

### Measures

Established and reliable tools were used for data collection. The APACHE II^[25]^ measured severity of illness within 24 h of ICU admission; the Santa Clara Strength of Religious Faith^[26]^ tool measured FSDM and MD religious beliefs; and the Dickenson scale^[27]^ measured MD attitudes related to the care of dying patients. MD and FSDM outcome expectations for survival, and goals of care were measured using questions and approaches employed in prior work^[28]^.

At each data collection point, MDs and FSDMs were asked a single item question regarding goals of care for the patient at that point in time. Goals of care was conceptually defined as “an understanding of what is most important to the patient in order to allow the clinician to align the care provided accordingly”^[29,30]^. Operationally, MDs were asked, “regarding medical decisions for your patient, what is most important to you right now?” using a visual analog scale anchored at “Comfort/quality of life” at one end and “Survival, length of life” at the other. The goal of care was represented numerically with values of 50 representing equal weight placed on goals of survival and QOL. Values >50 represented more weight placed on a goal of survival while values <50 represented more weight placed on a goal of QOL. MDs were also asked at each time point, “regarding medical decisions for your patient, what do you think is most important to the FAMILY right now?” using the same visual analog described above. FSDM were also asked “regarding medical decisions for your loved one, what is most important to you right now?” using the same visual analog scale.

Physician and family outcome expectations for survival were measured by asking each subject to indicate expectations for 3 months in the future for survival (very unlikely/not likely vs. very likely/likely) - a similar approach used in other work^[31]^. Data regarding actual survival were obtained for all subjects at the end of the study using chart abstraction.

### Procedure

Research assistants (RA’s) made rounds every day in the three study ICUs and identified eligible patients. Data were collected every 5 days, until ICU discharge or patient death. Physician data were collected within 24 h of the time of family data collection. Because there was on-going variation in the physicians who were caring for the patient on the day of data collection, each physician was assigned a unique identification number in order to allow analysis of the influence of the rotation pattern on changes in focus of care over time.

### Data Analysis

Power analysis conducted for the main study analyses indicated that a sample size of 236 with an alpha of 0.05 had a power=0.995^[32]^. Frequencies and measures of central tendency were used to describe the sample. For group comparisons, one-way analysis of variance (ANOVA) tests for continuous variables and χ^2^ for categorical variables were employed.

Bivariate correlations were conducted for examination of relationships between continuous variables, and multiple regression analysis was used to examine possible predictors of MD-FSDM discordance in goals of care. For all analyses, a two-sided p value <0.05 was considered to be statistically significant. All analyses were performed using SPSS Version^[24]^.

## RESULTS

Demographic and clinical characteristics for patients (n=264), family surrogates, and physicians are shown in Table 1. As seen in Table 1 patients had an average ICU stay of 2 weeks with an additional one week stay in the hospital. Fewer than half had any documentation of advance directives at admission and 27.3% died during their hospital stay. An additional 45 subjects died during the first 3 months post-hospital discharge-30 died within 1 month of hospital discharge, an additional 7 died between one and two months after hospital discharge, and 8 died between two and three months of hospital discharge. This yielded an overall 3 month post-hospital mortality rate of 43.9% for the entire sample.

**Figure 1:**
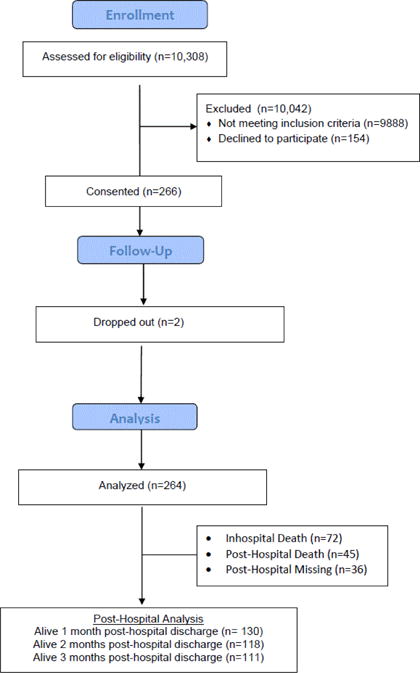
CONSORT diagram: Subject enrollment.

Patient admissions were approximately evenly distributed between Medical ICU (MICU), Surgical ICU (SICU), and Neurologic ICU (NICU). Physicians had an average of 6 years of clinical practice in the ICU, were primarily an attending MD or fellow. Family surrogate decision makers (FSDMs) were predominantly female, the spouse of the patient, and employed. Figure 1 displays the enrollment and attrition data for the study.

First, we compared clinical and demographic characteristics of patients who died to patients who survived the study period. There were 116 deaths (43.9%) of study subjects during the study period with a majority of deaths (62.1% of all deaths) occurring during the hospital stay. Subjects who died were, on average, older (64.7 vs. 58.4 years, P=0.001), had a shorter hospital length of stay (20.1 vs. 24.2 days, P=0.027), had higher APACHE II scores (23.3 vs. 19.1, P<0.001), and higher comorbidity scores (4.9 vs. 3.5, P<0.001) than those who survived.

### Prognostic Expectations for Mortality

**Figure 2:**
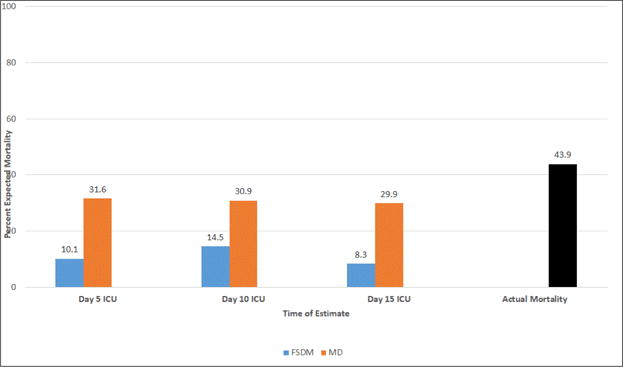
Mortality predictions over time (n=253, n=177, n=77, respectively).

Next, we examined prognostic expectations over time for mortality by MDs and FSDMs. As noted earlier, prognostic expectations were dichotomous: survival was prognosticated to be either very unlikely/not likely (survival predicted) or very likely/likely (mortality predicted). As seen in Figure 2, MDs had higher expectations of patient mortality than did FSDMs at all points in time. However, MD predicted mortality was lower than actual mortality at every time point. Unlike MDs, FSDMs expectations varied significantly over time. The changes in mortality expectation by FSDMs between time points were statistically significant (Time 1 and 2: φ=0.57, P<0.001; Time 2 and 3: φ=0.50, P<0.001).

Next, in order to further explore the predictive accuracy of MDs and FSDMs, we employed metrics used by others who have examined MDs and other mortality predictive measures^[33]^. MD sensitivities ranged from 46% to 51% and specificities ranged from 80% to 84%. FSDM sensitivities ranged from 18% to 22% and specificities ranged from 91% to 100%. As seen in Table 2, DORs (+LR/−LR) were greatest at Time 1 for both MDs and FSDMs.

Finally, we examined discordance between MDs and FSDMs regarding their prognosis for mortality over time. Discordance was 79.7% at Time 1 (φ=0.24, P<0.001), 67.4% at Time 2 (φ=0.34, P<0.001) and 82.4% at Time 3 (φ=0.27, P=0.04).

### Goals of Care

**Figure 3:**
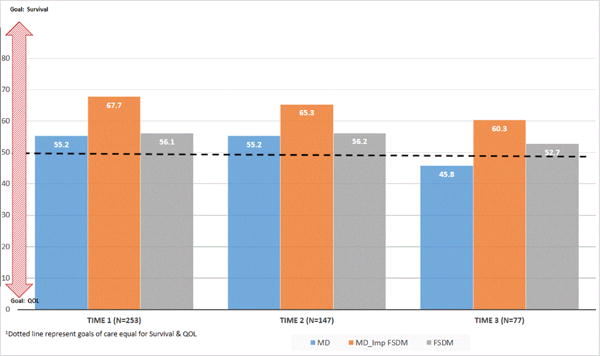
Mean goals of care for MD, what MD thinks is important to FSDM and mean goals of care for FSDM.

Next we examined the goals of care that the MD had for the patient, the goals of care that the FSDM had for the patient, and the goals of care that the MD thought that the family wanted for the patient. As seen in Figure 3, FSDMs’ goals of care did not vary much over time (p=0.45) while the MDs goals of care moved towards QOL (values <50) over time (P=0.002, partial η^2^=0.10). MDs goals of care were significantly different from Time 1 compared to Time 3 (mean difference 10.9, 95% CI: 1.36–20.36, P=0.02) and Time 2 compared to Time 3 (mean difference 11.99, 95% CI: 3.76–20.21, P=0.002). At Time 1 the difference between FSDM goals and what the MD thought the FSDM goals were was, on average, −12.16 (SD: 39.51), 95% (CI: −17.22–7.11), P<0.001. The difference at Time 2 was −8.89 (SD: 43.96), 95% CI: −16.55–1.23), P=0.023 and at Time 3 the difference was −9.44 (SD: 40.05), 95% CI: −19.97–1.09, P=0.078.

We were also interested in examining the relationship between FSDMs characteristics (age, race, gender, religious faith, relationship to patient, evaluation of effectiveness of treatment, FSDM expectation of survival) and MDs expectation of survival with FSDM goals of care at each point in time. At Time 1 the sole variable that significantly related to goals of care of the FSDM was the FSDMs’ expectations of the patient’s survival (β=0.27, P<0.001). At Time 2, both FSDMs’ expectations of the patient’s survival (β=0.30, P=0.002) and MDs’ expectations of the patient’s survival (β=−0.19, P=0.048) related to the goals of care of the FSDM. At Time 3, there were no variables that had significant relationships with FSDM goals of care.

### Goals of Care: Discordance between MDs and FSDMs

Finally, we were interested in examining and describing characteristics of MD-FSDM discordance between goals of care over time. We defined discordance as being a difference between the MD and FSDM’s goal of care (ranging from 0 for most weight placed upon QOL to 100 representing most weight placed upon survival) with positive values representing a situation where the MD desired survival more than the FSDM. We classified discordance as an absolute difference of >40 in MD and FSDM goals of care. We chose to use a difference of 40 points because that difference would indicate a significantly different weighting of what was important to the two respondents (MD and FSDM). The difference of 40 would result either from the respondents placing the goal at different ends of the scale (QOL vs. survival) or, in some cases, indicating that for one respondent, one goal was all that mattered (i.e., 90–100), while the other respondent indicating that survival and QOL were almost of equal importance (i.e., 40–60).

MD-FSDM discordance in goals of care increased over time with rates of 36.4%, 42.6% and 55.4% of dyads being discordant at Time 1, 2 and 3, respectively. Only the difference in discordance at Time 1 (36.4%) compared to Time 3 (55.4%) was significantly different (P=0.003). When examining only those cases identified as discordant, we found that at Time 1, 52.3% of the cases classified as discordant represented situations where FSDMs wanted more survival than did MDs. This trend remained stable over time with 52.7% and 58.1% of FSDMs wanting more survival than did MDs at Times 2 and 3, respectively; none of the differences over time were statistically significant.

**Figure 4:**
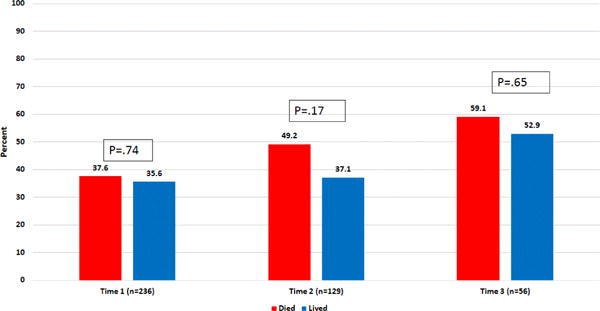
MD-FSDM discord by mortality outcome overtime.

Next, we examined whether there were different rates of discordance by mortality outcome. As seen in Figure 4, at each point in time, for patients who died, there was a higher rate of discordance than for patients who lived, but these differences were not statistically significant (Figure 4). When examining changes in discordance over time, for those who lived, there was a significant increase in discordance rates from Time 1 to Time 3 (difference=16.38%, P=0.034) but not from Time 2 to Time 3 (difference=15.80%, P=0.057) or from Time 1 to Time 2 (difference 1.5%, P=0.78). For those who died, there was a significant change in rates of discordance from Time 1 to Time 3 (difference=21.47%, P=0.038) but not a significant change in rate from Time 1 to Time 2 (difference=11.53%, P=0.068) nor from Time 2 to Time 3 (difference=9.94%, P=0.35).

Finally, we were interested in examining whether there were significant differences in MD or FSDM characteristics (age, gender, race, relationship to patient, APACHE score) that related to the presence or absence of discordance. There were no statistically significant differences between those with and those without MDs and FSDMs discordance.

## DISCUSSION AND CONCLUSION

Decision making for CCI patients is complex and can be difficult. While the importance of discussing goals of care when caring for patients with complex illnesses has been established^[21,34,35]^. There remains difficulty in implementing such discussions-especially in the ICU setting. In this study, we have reported several findings of note.

First, we found that MDs consistently under-estimated mortality rates over time (large effect size) and that there was a high rate of discordance between MDs and FSDMs regarding mortality prognosis (medium effect size). Our rates of discordance about prognosis are higher at all points in time when compared to those reported by others examining family and MD prognosis of mortality in the ICU setting^[19]^. In addition, our findings support prior work that has demonstrated variability and inaccuracy of MDs in terms of predicting mortality^[3,31,33]^.

Not surprisingly, family expectations were more unrealistic than those of physicians-a finding supported by others^[19,22]^. To the extent that expectations are a key component of family decisions regarding giving or withholding permission for limitations on aggressiveness of interventions (e.g. tracheostomy, dialysis, etc.), overly optimistic expectations can be associated with prolonged use of ineffective therapies. In addition, the medium effect size associated with the discord between FSDMs and MDs in terms of mortality predictions reinforces other work that has focused upon communication barriers between MDs and family regarding outcomes of care and treatment decisions^[3,19,36–38]^.

Secondly, we found evidence that the MD and FSDM were not aligned regarding goals of care for the patient and that MDs consistently favored survival over a QOL goal-an emphasis that was sometimes not what the FSDMs had identified as important to them. This finding is consistent with our previous analysis that demonstrated that survival expectation was the strongest force associated with aggressive treatment plans focused on survival and that other factors such as age, race and religiosity did not influence FSDMs’ survival expectations^[28]^. Missed opportunities to communicate, ineffective or inconsistent communication, and misunderstanding about key issues such as goals of care all may lay the foundation for care that is more aggressive than what is desired by the family-an identified source of dissatisfaction by family of ICU patients^[22,36–40]^.

Finally, we were able to examine goals of care and discordance over time (from 5 days in the ICU to 15 days in the ICU) and found that discordance rates increased over time. Thus, more time spent in the ICU was not associated with better consensus between MDs and FSDMs regarding mortality expectations or goals of care. There could be many explanations for this. First, some family members have enormous emotional barriers to recognizing the impending loss of a loved one, despite best efforts of clinicians to prepare them. In these cases, even if the clinical staff are recognizing that lack of improvement in the patient’s condition over time suggests a poor outcome, family members may hold on to hope, and thus the discordance in expectations and goals can increase. Second, we were not able to control for the influence of communication from the many other clinicians caring for the patient, and thus families may have been receiving more hopeful messages from others, such as consultants. Third, given the relatively low rate of advance directives present, it is possible that some families were influenced by their beliefs about what the patient would want, which might have been different than their own preferences and values.

While our data do not include evaluations of the communication, these results do suggest that we must continue to identify approaches that can more effectively and efficiently lead to better understanding between treatment teams and FSDMs if we are reduce the use of ineffective interventions and improve the care of patients at the end of life^[3,19,22]^. Thus, we have reason to believe, based upon our findings, that there is a lack of meaningful communication between MDs and FSDMs that may be exacerbated over time. This lack of communication regarding relevant issues (mortality, goals of care) coupled with poor estimates of mortality (by MDs and FSDMs) may serve as significant barriers to making treatment decisions that reflect wishes of families, pursuing palliative care and in some cases, facilitating end of life discussions. Until we ask the family what their expectations are for mortality and what their goals of care are (two basic and easily answered questions), we will continue to be ill equipped to facilitate discussions and decisions that lead to meaningful decisions for these prognostic expectations. One approach to enhancing communication would be to have a central form (or practice) for asking MDs, RNs and family decision makers to assess their goals of care (on a Comfort Only–Survival Only continuum) as well as assess their expectations for survival. This would provide both healthcare providers and family decision makers with key information that could facilitate additional discussions regarding goals of care and treatment decisions.

This study had several strengths. First, we interviewed MDs and family members in “real time” and were able to conduct a longitudinal (not cross sectional) study. As a result, we were able to examine changes in prognostication and goals of care over time and enhance the clinical relevance of our findings. Second, we had a cohort of family subjects that were ethnically diverse and who represented different ICU environments and physician groups. Thus, the generalizability of our study results was strengthened. Finally, we also had a diverse sample of physicians in terms of ethnicity, years of employment and age. This diversity also serves to facilitate in having a more representative sample and in being able to more fully describe variables of interest.

Our study had some limitations. First, responses to value-laden questions about what was important may reflect response bias and the influence of social desirability. That is, both physicians and family members may have been reluctant to indicate values that could be seen as inadequately recognizing the importance of quality of life. Importantly, we confined our data collection to the intensivists with whom the family members had the most routine contact.

Second, there is the possibility that there were influences from others (not included in our study) that may have had an impact upon the responses provided by families and MDs. For example, strong influence from other physicians, particularly the patient’s surgeon may have had an impact on individual MDs assessments of mortality or goals of care. Similarly, other family members likely influenced the decisions of the primary decision maker, and these sources of influence could not be readily measured.

Despite these limitations, we believe that this study provides important insights into the dynamic process of decision making for chronically critically ill patients in the real world and over time. Without standard processes-endorsed by healthcare providers–to assure that values of patients and families are solicited and incorporated, it will be difficult to reduce the discordance between physicians and family surrogates regarding goals of care and treatment decisions that continue in the ICU.

## Figures and Tables

**Table 1:** Sample description.

Variable	Mean (SD)	N (%)
**Patient (n=264)**		
Age (years)	61.2 (15.4)	
APACHE II score^1^	20.1 (8.7)	
Charlson Comorbid Score^2^	4.1 (2.7)	
Intensive Care Unit (ICU) stay (days)	14.0 (8.2)	
Hospital stay (days)	22.4 (14.7)	
Length of mechanical ventilation	10.9 (8.2)	
Race: Caucasian	56.4 (29.1)	
Gender: Female		177 (67.3)
Admitting ICU Service:		119 (45.1)
Medical ICU		92 (34.8)
Surgical ICU		78 (29.5)
Neurologic SU		94 (35.6)
Living Will @ Admission: Yes		89 (33.7)
Durable Power of Attorney @ Admission: Yes		106 (40.3)
Do Not Resuscitate Order @ Admission: Yes		36 (13.7)
Died in-hospital: Yes		72 (27.3)
Died at any point during study period: Yes		116 (43.9)
**Physician (n=54)**		
Age (years)	36.0 (7.9)	
ICU Practice (years)	6.1 (6.8)	
Santa Clara Score (Sense of Faith)^3^	11.6 (4.5)	
Dickinson Item 2: Comfort with Dying Patients^4^	2.0 (1.1)	
Dickinson Item 7:Comfort with Family of Dying Patients^5^	2.5 (1.0)	
Race: Caucasian		34 (64.2)
Gender: Female		15 (28.3)
Clinical Status: Attending or Fellow		46 (86.7)
**Family Surrogate Decision Maker (n=264)**		
Age (years)	55.3 (13.3)	
Health Status^6^	3.4 (1.0)	
Santa Clara Score (Sense of Faith)^3^	16.1 (3.7)	
Race: Caucasian		176 (66.7)
Gender: Female		202 (76.5)
Relationship to Patient: Spouse		107 (40.5)
Employed: Yes		151 (57.2)
Decrease/Loss Employment Since Patient Illness: Yes		100 (65.8)
Household Income: >$50,000		106 (45.7)

NOTE:

1 Range: 0–71 (higher=higher severity of illness);

2 Scores >5 indicate high risk of 1 year mortality;

3 Range: 0–40 (higher=stronger sense of faith);

4 Range: 1–5 (higher=more comfort with caring for dying patient;

5 Range: 1–5 (higher=better physical health status)

**Table 2:** Accuracy of mortality predictions by ICU physicians and FSDM of CCI patients.

	Sensitivity	Specificity	Positive Likelihood Ratio^2^	Negative Likelihood Ratio^3^	Diagnostic Odds Ratio^4^
Time 1: MD	0.51	0.84	3.22	0.57	5.65
95% CI	0.42–0.62	0.77–0.89	2.13–4.88	0.47–0.71	
Time 2: MD	0.46	0.8	2.31	0.67	3.45
95% CI	0.34–0.59	0.70–0.88	1.41–3.77	0.53–0.86	
Time 3: MD	0.5	0.8	2.55	0.62	4.11
95% CI	0.30–0.70	0.40–0.72	1.30–5.01	0.41–0.93	
Time 1: FSDM	0.18	0.97	5.27	0.85	6.2
95% CI	0.12–0.27	0.92–0.99	2.05–13.54	0.77–0.93	
Time 2: FSDM	0.22	0.91	2.41	0.86	2.8
95% CI	0.12–0.34	0.82–0.96	1.03–5.68	0.74–1.00	
Time 3: FSDM	0.21	1	Cannot be calculated^1^	0.79	-----
95% CI	0.07–0.42	0.93–1.00		0.64–0.97	

1 Cannot be calculated because specificity=1.0

2 Likelihood Ratio+ (LR+)=Sensitivity/1 - Specificity

3 Likelihood Ratio- (LR−) =1 - Sensitivity/Specificity

4 Diagnostic Odds Ratio=LR+/LR− [measure of effectiveness of a diagnostic test with binary classification]
